# EXPLORING THE RELATIONSHIP BETWEEN RELIGION, SPIRITUALITY, LONELINESS, SOCIAL ISOLATION, AND AGING

**DOI:** 10.1093/geroni/igae098.0629

**Published:** 2024-12-31

**Authors:** Roger O’Sullivan, Katherine Britt, Jim Lubben

**Affiliations:** Chair; Co-Chair; Discussant

## Abstract

The importance of social engagement, connections, and participation as we age and the negative impact of loneliness and social isolation on health are well recognized. Religion and/or spirituality has social, emotional, and existential dimensions and are linked to concepts of belonging, meaning, and purpose. As part of the drive to build meaningful social and emotional connections in contemporary society, religion and/or spirituality are proposed as potential solutions to loneliness/social isolation. The theoretical mechanism proposed is that affiliation, attendance, practice, or belief can offer support, social capital, meaning and a sense of belonging. This interdisciplinary symposium uses data from both the US and Europe to explore the relationship between religion, spirituality, loneliness, and social isolation. Leavey using longitudinal data from Ireland explores depression and anxiety and their relationship with loneliness, social networks, religious practice, and long-term illness. Victor & Seetal, also using longitudinal data set out the relationship between measures of religious affiliation, attendance, and practice with loneliness for older adults in England. Taylor uses the HRS to examine the association between religious service attendance and spirituality on social isolation and loneliness among older adults in the United States. Finally, Hampton Jarmon, using data from the Healthy Aging in Neighborhoods of Diversity Across the Life Span study, explores the relationship between spirituality (connectedness, prayer fulfilment, and universality) and cognition within Black and White adult participants. Lubben, as discussant, will reflect on the key themes and the symposium’s overarching focus - can religion and/or spirituality provide protection from loneliness and social isolation?

